# The Quality of Services of Iran University Hospitals Based on SERVQUAL's Evaluation Model: A Systematic Review and Meta-Analysis

**DOI:** 10.3389/fpubh.2022.838359

**Published:** 2022-04-18

**Authors:** Abdolreza Gilavand, Amin Torabipour

**Affiliations:** ^1^Department of Education Development Center, Ahvaz Jundishapur University of Medical Sciences, Ahvaz, Iran; ^2^Department of Health Services Management, School of Health, Ahvaz Jundishapur University of Medical Sciences, Ahvaz, Iran

**Keywords:** medical education, health policy, quality measurement, SERVQUAL, Iran

## Abstract

**Introduction:**

Systematic evaluation of the quality of services provided in hospitals and healthcare centers is the first step toward standardization and improving their quality.

**Methods:**

In this systematic review (meta-analysis) study, the information was collected by searching for the articles published in well-known Iranian and international and through searching for the keywords of SERVQUAL, services quality, gap, hospital, patients, Iran, and without language and time restrictions.

**Results:**

A total of 18 articles were reviewed and opinions of 4,714 people who referred to hospitals and healthcare centers affiliated to Iran University of Medical Sciences during the past 10 years from 2010 to 2019 were examined in this study. The results showed that there was a quality gap in all 5 dimensions between the current status and desirable status of patients and based on a maximum score of 5, responsiveness (1.04), and assurance (0.99), empathy (0.95), reliability (0.91), and physical or tangible factors (0.86) were ranked first to fifth, respectively. According to the random effect model, the mean score of patients' perceptions and expectations and the gap between them was 3.59 (CI 95%: 3.73, 3.46), 4.66 (CI 95%: 4.33 and 4.66), and 0.86 (CI 95%: 1.00, 0.72), respectively.

**Conclusion:**

The patients' expectations in university hospitals were higher than their perceptions. Therefore, it is recommended for Ministry of Health and Medical Education of Iran to monitor periodically the quality of hospitals while focusing on students' and patients' satisfaction and pay attention to dimensions that have the highest quality gap.

**Systematic Review Registration:**

https://ethics.research.ac.ir/ProposalCertificateEn.php?id=167856&Print=true&NoPrintHeader=true&NoPrintFooter=true&NoPrintPageBorder=true&LetterPrint=true, identifier: IR.AJUMS.REC.1399.747.

## Introduction

Nowadays, improving the quality of healthcare service provision to meet the expectations of patients and to satisfy them, has become a major challenge for service providers (1). Healthcare systems are under increasing pressure to meet and respond to ever-increasing public demands and demographic changes to improve their performance, and as the service risk of these organizations is high, the provided services should have an acceptable quality (2). Service quality can be used as a strategic difference to create competitive advantage (3). Systematic evaluation of the quality of services provided in hospitals and healthcare centers is the first step toward standardization (national standardization and international standards), enhancement and improvement of their quality. In the hospital setting, patients are the most important group for evaluating the quality of hospital services (4). The traditional approach to evaluate the quality of healthcare service emphasized the use of objective criteria such as mortality rates and prevalence of diseases. Although these indicators are essential for evaluating the quality of clinical services, subjective evaluations are commonly used nowadays (5). The SERVQUAL's evaluation model, the quality of provided services is determined by examining the gap between expectations (desirable status) and perceptions (current status) based on five dimensions such as physical dimension, assurance dimension, responsiveness dimension, reliability dimension, and empathy dimension. This model has been used in several studies to evaluate the quality of hospital healthcare services provision (6, 7). For example, in recent years, the SERVQUAL model has been used to measure patients' satisfaction with the quality of healthcare services provided in Turkish hospitals (8, 9), Saudi Arabia (10, 11), Poland (12), Cyprus (13), Albania (14), India (15, 16), Pakistan (5, 17) and Ghana (18). In Iran, which has a unique healthcare service provision system, several studies have been conducted (19–40). In Iran, the medical education system and the healthcare service provision system in 1985, was integrated and resulted in establishment of a new Ministry under the title Ministry of Health and Medical Education (MOHME) (41, 42). Each independent university or faculty of medical sciences within its geographical area is responsible for teaching different medical fields and providing healthcare services. In this system, which is exclusive to Iran (43).

Many studies have been conducted individually and regionally to evaluate the quality of services provided in hospitals affiliated to Iran universities of medical sciences, but no systematic review has been conducted so far. As a systematic evaluation of the quality of services provided in hospitals and healthcare centers is the first step toward standardizing, promoting and improving their quality, this study investigates the quality of healthcare services provision in hospitals affiliated to Iran University of Medical Sciences. In addition to evaluating the quality of healthcare services provided by Iran's university hospitals, this study can also help us more clearly determine whether this unique experience has been successful in Iran and can be used by other countries.

## Data Sources

This systematic review (meta-analysis) study was conducted in January 2020. The research data was collected by searching for articles published in well-known Iranian and international sites (including SID, MAGIRAN, Iranmedex, GoogleScholar, Embase, PubMed, Scopus, Doaj, Science Direct) and by searching for the keywords SERVQUAL, healthcare services, hospital and Iran with no language or time restrictions. Endnot X5 software was used for organizing the references and eliminating duplicate and similar studies. Additional articles were also searched manually to find review articles on evaluating the quality of hospital healthcare services and to list the reference of articles that met the inclusion criteria.

## Study Selection

**Figure 1:**
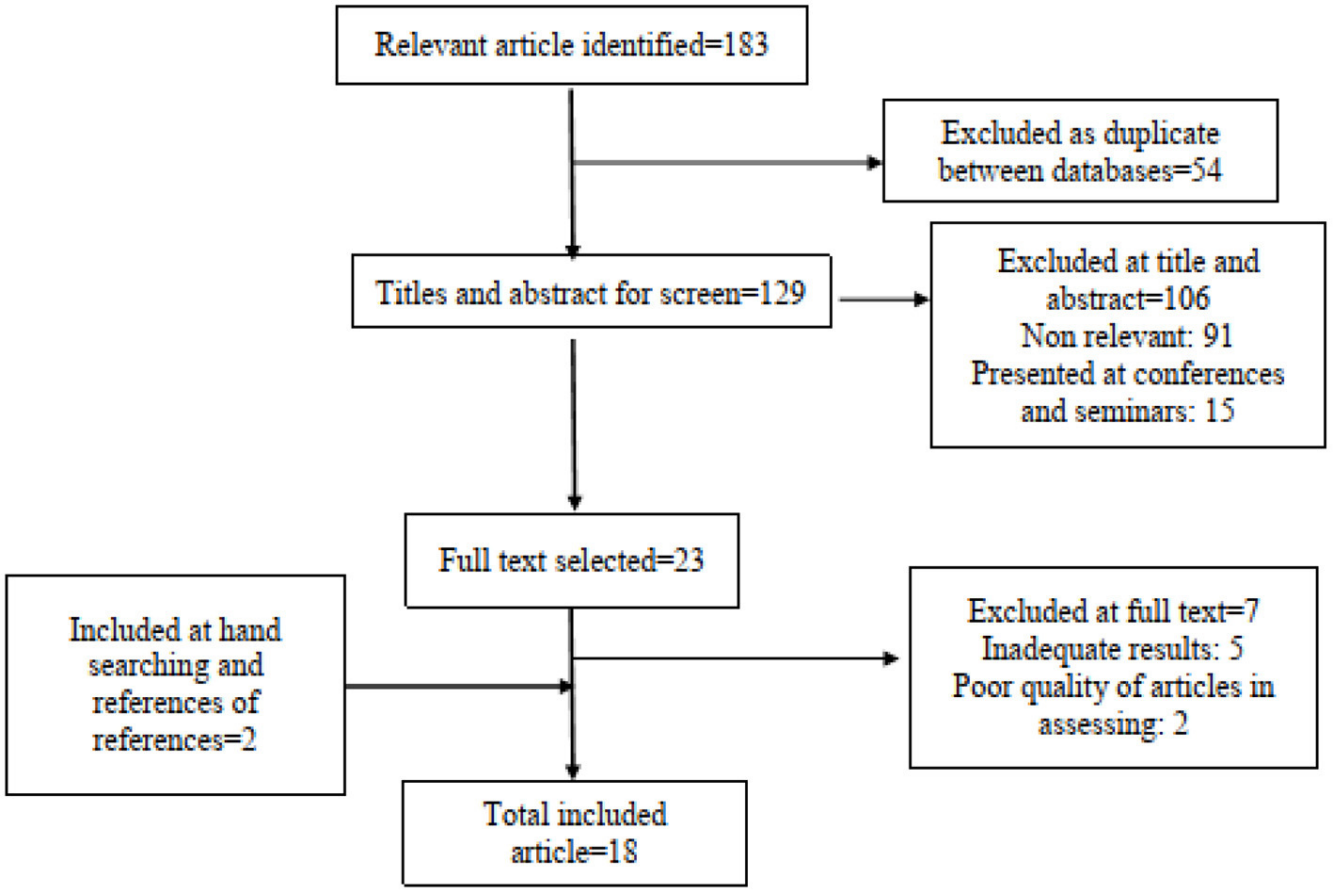
Flow diagram for study selection.

Abstracts and titles of all studies were also independently reviewed to identify major studies evaluating patients' perceptions of the quality of provided healthcare services. The articles were considered acceptable and selected if they met the following criteria: Original research, conducted in one of the hospitals affiliated to one of the medical universities of Iran, reporting the mean score of patients' perceptions and expectations of the quality of services received, without language and time restrictions. Also, the articles under preparation, case reports, and interventional studies were considered as non-acceptance criteria in this study and were excluded from the review process. However, two articles that did not report standard deviation and were not calculable were excluded from the meta-analysis process (Figure 1).

## Review of Quality

The articles were evaluated by two reviewers individually based on the 'strengthening the report of observational studies in epidemiology' (STROBE) checklist. A third reviewer was used to resolve the difference of opinions between the two reviewers.

## Data Extraction

In the next stage, the two reviewers collected data from existing articles using a standard form. For each study, information about survey characteristics including author, year of study, study context, sample size, mean score of 5 dimensions of SERVQUAL questionnaire and important findings were extracted.

## Data Analysis

In this systematic review (meta-analysis) study, STATA version 16 software was used to estimate the mean service quality score. *I*^2^ index was also used to evaluate the heterogeneity of the studies. Since heterogeneity was also found among the studies (except for the mean perception in the physical dimension using the fixed effects model), the random effects model (*P* value of Q > 0.05 or *I*^2^ > 50%) was used with a 95% confidence interval. Since only 50% of the publication bias is detectable in the funnel plot, the Egger test was used to evaluate the probability of the publication bias. Significance level for the indicators of total mean perceptions, total mean expectation, and total gap were calculated at *p* = 0.895, *p* = 0.007, and *p* = 0.908, respectively, indicating that the probability of publication bias was not statistically significant in total mean perception and gap.

## Results of Data Synthesis

To evaluate the quality of healthcare services provided by Iran Medical Sciences Universities based on SERVQUAL evaluation model, out of 183 existing studies, a total of 18 studies were found to be eligible for systematic review and meta-analysis. Finally, the opinions of 4,714 people who referred to hospitals and health centers affiliated to Iran University of Medical Sciences during the past 10 years from 2010 to 2019 were reviewed. Among them, 6 were written in English language and 12 were in Persian language (according to Table 1).

**Table 1:** Specifications of selected articles.

	**Authors**	**Year of implementation**	**University of Medical Sciences**	**Number of patients**	**Article language**
1	Vazifeh and Estanesti (19)	2019	Zahedan University of Medical Sciences	96	Persian
2	Qolipour et al. (21)	2018	Ahvaz Jundishapur University of Medical Sciences	250	English
3	Hashemi et al. (22)	2018	Kerman University of Medical Sciences	83	Persian
4	Vizvari et al.(23)	2018	Golestan University of Medical Sciences	175	English
5	Razmjoee et al. (24)	2017	Shiraz University of Medical Sciences	98	English
6	Haghshenas et al.(25)	2017	Tehran University of Medical Sciences	225	Persian
7	Rezaei et al. (26)	2016	Kermanshah University of Medical Sciences	400	English
8	Kazemnezhad et al. (27)	2016	Qom University of Medical Sciences	409	Persian
9	Aghamolaei et al. (29)	2014	Hormozgan University of Medical Sciences	96	English
10	AbolghasemGorji et al. (31)	2013	Tehran University of Medical Sciences	116	Persian
11	Ameryoun et al. (32)	2013	Tehran University of Medical Sciences	264	Persian
12	Razlansari et al. (30)	2012	Kerman University of Medical Sciences	400	Persian
13	Mohammadi and Mohammadi (33)	2012	Zanjan University of Medical Sciences	300	English
14	Tarrahi et al. (34)	2012	Lorestan University of Medical Sciences	650	Persian
15	Tabibi et al. (35)	2012	Tehran University of Medical Sciences	242	Persian
16	Jenaabadi et al. (40)	2011	Zahedan University of Medical Sciences	200	Persian
17	Havasbeigi et al. (37)	2013	Kermanshah University of Medical Sciences and Ilam University of Medical Sciences	450	Persian
18	Hekmatpou et al. (38)	2010	Arak University of Medical Sciences	260	Persian
Total				4,714	

**Figure 2:**
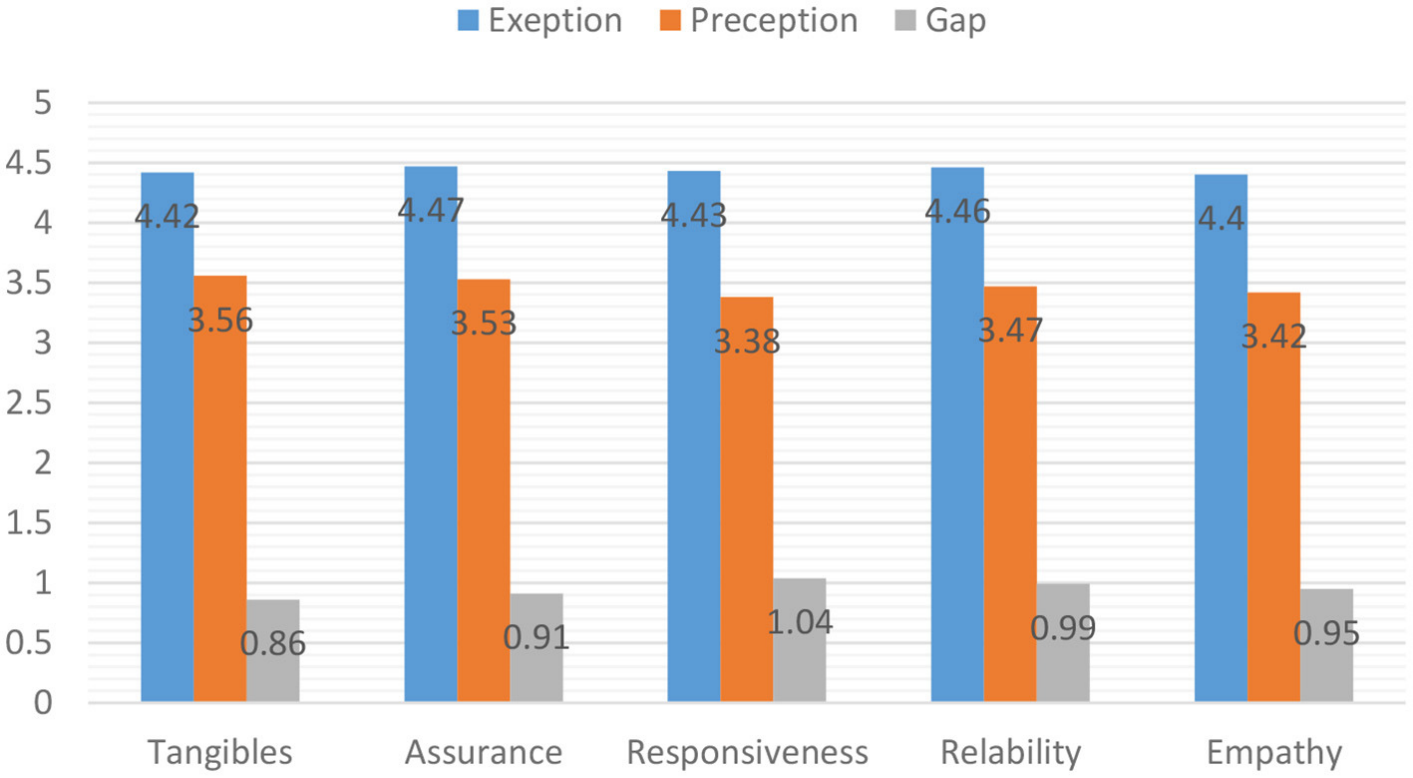
The mean score of patients' perceptions, patients' expectations, and gap between them (Reliability is misspelled in the Figure above).

Based on the random effect model, the mean score of patients' perceptions, patients' expectations, and gap between them was 3.59 (CI 95%: 3.73 and 3.46), 4.50 (CI 95% 4.66, 4.33), and 0.86 (CI 95%, 0.72, 1.00) 0.86, respectively. The largest gap was related to the dimension of responsiveness (as shown in Figure 2).

Concerning the score of patients' perceptions of the quality of healthcare services, the highest score belonged to the study conducted in hospitals of Zahedan University of Medical Sciences in south eastern Iran with a mean score of 4.18 (40) and the lowest score belonged to the study conducted in the university hospitals of Ilam and Kermanshah provinces in western Iran with a mean score of 2.78 (37). In the section of expectations from quality of healthcare services, the highest mean belonged to the study performed in hospitals affiliated to Arak University of Medical Sciences in central Iran (38) and the study performed in Zahedan University of Medical Sciences in southeastern Iran with a mean a score of 4.95 (40), and the lowest score belonged to a teaching hospital affiliated to Kerman University of Medical Sciences in central Iran with a mean score of 3.93 (22). Also, the highest gap was observed in studies conducted in health centers affiliated to Qom University of Medical Sciences in central Iran with a mean score of 1.39 (27) and the lowest gap ratio belonged to hospitals affiliated to Golestan University of Medical Sciences in northern Iran with a mean score of 0.31 (23). Concerning the five dimensions of quality, the analysis of the results of the review studies in terms of patients' perceptions showed that the highest score belonged to the physical dimension of quality (3.56) and the lowest score belonged to the dimension of responsiveness (3.38). Also, in the section of expectations of quality of healthcare services, the highest score belonged to assurance dimension (4.47) and the lowest score belonged to empathy dimension (4.40).

## The Physical or Tangible Dimension

In different studies, the highest mean perception in physical dimension belonged to health centers affiliated to Lorestan University of Medical Sciences in western Iran with a mean score of 3.93 (34) and the lowest mean perception belonged to health centers affiliated to Qom Medical University of Science in central Iran with a mean of 2.91 (27). Meta-analysis or merged estimate of mean effects in a result of study reviewed 16 articles was obtained at 3.23 (CI 95%: 3.54, 3.19).

The highest mean expectation was reported in physical dimension in hospitals affiliated to Arak University of Medical Sciences (4.96) (38) and the lowest mean expectation was reported in university hospitals of Ilam and Kermanshah provinces with a mean of 4.00 (37). Meta-analysis or merged estimate of mean effects was obtained in a result of study which reviewed 16 articles where (CI 95% 4.68, 4.21) was obtained at 4.45.

## Reliability Dimension

The highest mean perception in reliability dimension was reported in health centers affiliated to Lorestan University of Medical Sciences in West Iran with a value of 4.34 (34) and the lowest mean was reported in health centers affiliated to Zanjan University of Medical Sciences in Central Iran with a value of 02.55 (33). Meta-analysis or merged estimate of mean effects obtained in the study reviewed 16 articles (CI 95%: 3.88, 3.11) was obtained at 3.49.

The highest mean expectation in reliability dimension was reported in hospitals affiliated with Arak University of Medical Sciences in central Iran with a value of 4.97 and the lowest mean was reported in the study conducted by Havas Beighi in university hospitals of Ilam and Kermanshah provinces in western Iran with a value of 3.95 (37). Meta-analysis or merged estimate of mean effects in the study reviewed 16 articles (CI: 95% 4.71, 4.22) was obtained at 4.47.

## Responsiveness Dimension

The highest mean of perception in the responsiveness dimension was reported in health centers affiliated to Lorestan University of Medical Sciences in West Iran with a value of 4.20 and the lowest mean perception was reported in health centers affiliated to Zanjan University of Medical Sciences in Central Iran with a value of 0.49 (33). Meta-analysis or merged estimate of mean effects in the study reviewed 16 articles (CI 95%: 3.71, 3.05) was reported at 3.38.

The highest mean expectation in responsiveness dimension was reported in hospitals affiliated to Arak University of Medical Sciences in central Iran with a value of 4.94 (38) and the lowest mean was reported in hospitals affiliated to Golestan University of Medical Sciences in northern Iran with a value of 3.80 (23). Meta-analysis or merged estimate of mean effects in the study of 16 articles (CI 95%: 4.63, 4.26) was reported at 4.44.

## Assurance Dimension

The highest mean perception in assurance dimension was reported in health centers affiliated to Lorestan University of Medical Sciences in western Iran with a value of 4.26 (34) and the lowest mean perception was reported in university hospitals of Ilam and Kermanshah provinces in west of Iran with a value of 2.67 (37). The meta-analysis or merged estimate of mean effects in a study reviewed 16 articles (CI 95%: 3.86, 3.17) was obtained at 3.51.

The highest mean expectation in the assurance dimension was reported in Arak University of Medical Sciences hospitals in central Iran with a value of 4.96 (38) and the lowest mean was reported in a teaching hospital affiliated to Kerman University of Medical Sciences with a value of 3.89 (22). The meta-analysis or merged estimate of mean effects in the study reviewed 16 articles (CI 95%: 4.66, 4.24) was obtained at 4.45.

## Empathy Dimension

The highest mean of perception in the empathy dimension was reported in the health centers affiliated to Lorestan University of Medical Sciences in western Iran with a value of 3.89 and the lowest mean was reported in university hospitals of Ilam and Kermanshah provinces in western Iran with a value of 2.72 (37). The meta-analysis or the merged estimate of mean effects in study reviewed 16 articles (CI 95%: 3.67, 3.07) was reported at 3.37.

The highest mean expectation in empathy dimension was reported in hospitals affiliated with Arak University of Medical Sciences in central Iran with value of 4.94 (38) and the lowest mean was reported in a teaching hospital affiliated to Kerman University of Medical Sciences with a value of 3.84 (22). The meta-analysis or merged estimates of mean effects in a study reviewed 16 articles (CI 95%: 4.67, 4.81) was obtained at 4.43.

## Total Mean Expectation

**Figure 3:**
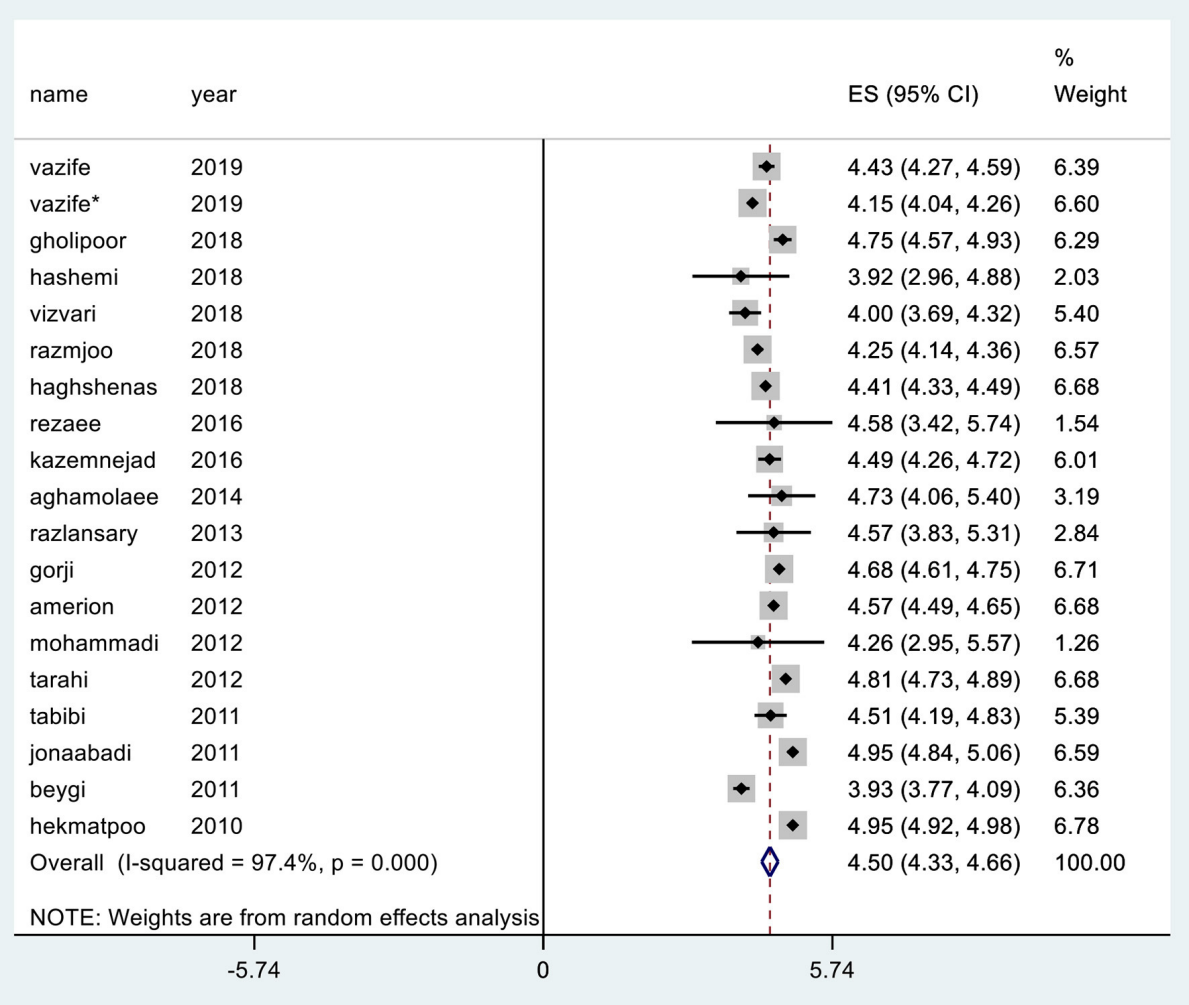
Mean expectation in the SERVQUAL questionnaire.

In various studies, the highest mean expectation was reported in hospitals affiliated to Arak University of Medical Sciences in central Iran with a value of 4.95 (38) and the lowest mean was reported in a teaching hospital affiliated to Kerman University of Medical Sciences with a value of 3.92 (22). Meta-analysis or merged estimate of mean effects in the study reviewed 18 articles (CI 95%: 4.66, 4.33) was obtained at 4.50 (Figure 3).

## Total Mean Perception

**Figure 4:**
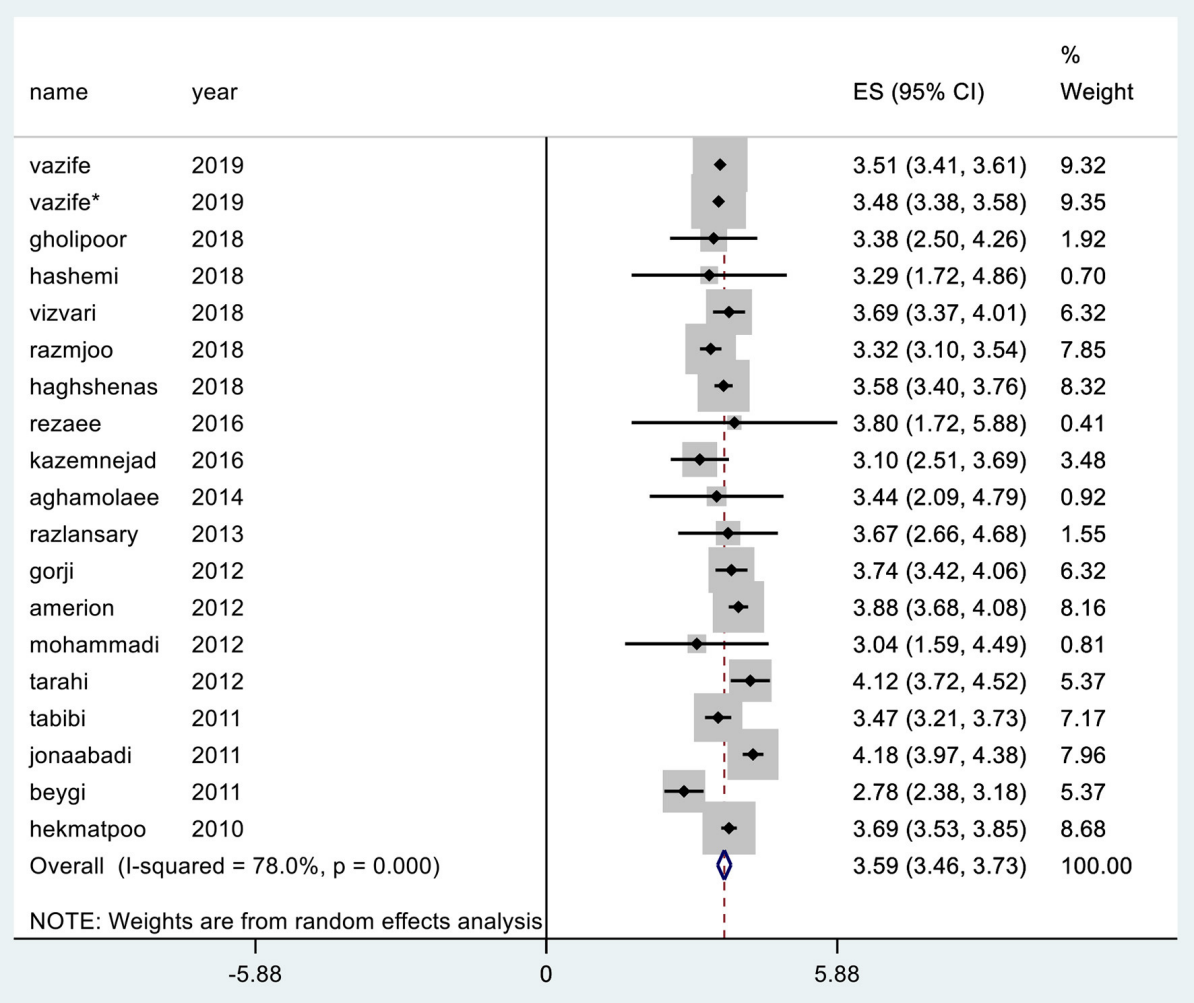
Total mean perception in the SERVQUAL questionnaire.

In various studies, the highest mean perception was reported in hospitals affiliated to Zahedan University of Medical Sciences in southeastern Iran with a value of 4.18 and the lowest mean was reported in university hospitals of Ilam and Kermanshah provinces with a value of 2.78 (37). Meta-analysis or merged estimate of mean effects in the study reviewed 18 articles (CI 95%: 3.73, 3.46) was obtained at 3.59 (Figure 4).

## Total Mean Quality Gap

**Figure 5:**
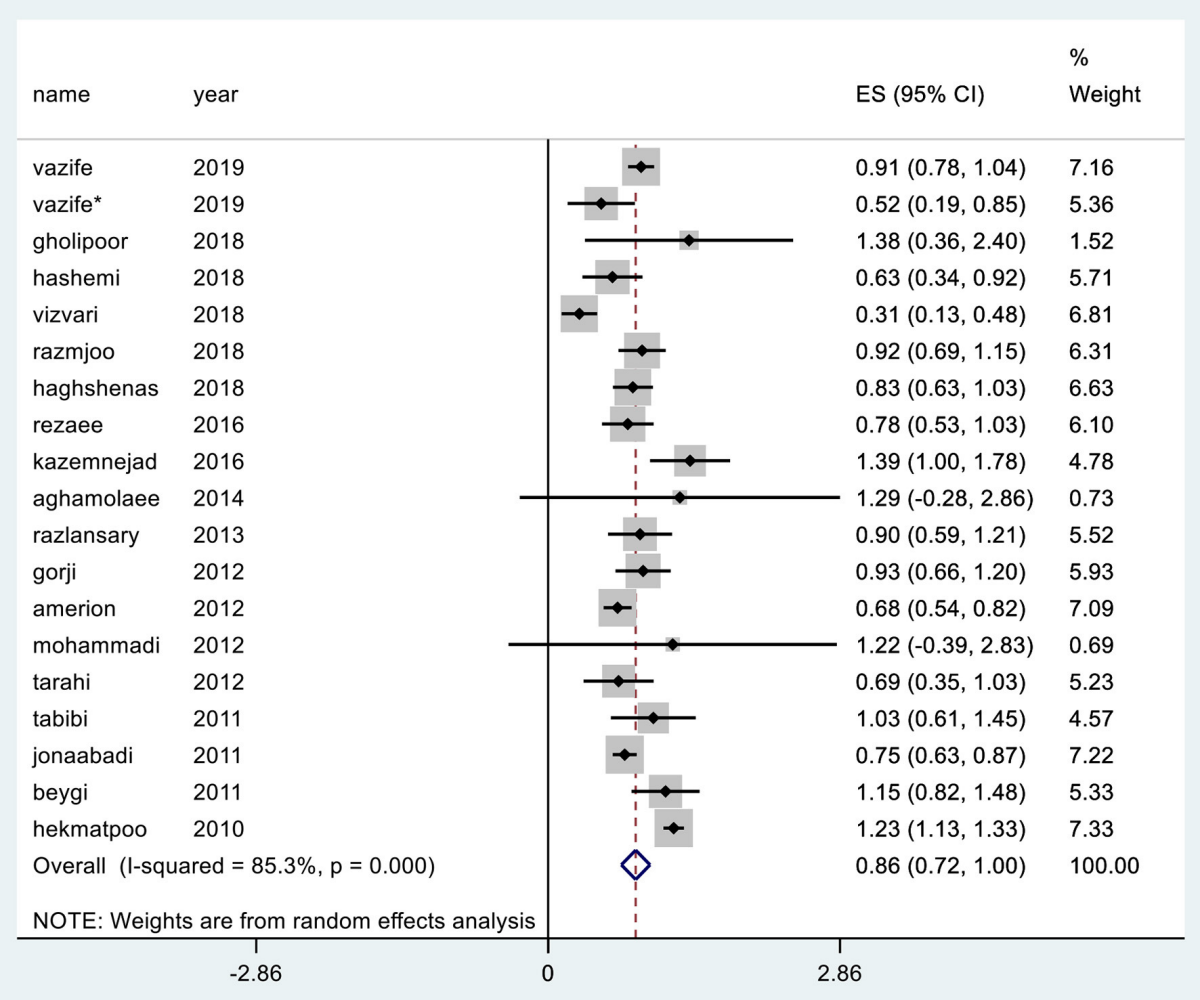
Total mean gap between perceptions and expectations of the SERVQUAL questionnaire.

In various studies, the highest mean gap was reported in health centers affiliated to Qom University of Medical Sciences in central Iran with a value of 1.39 and the lowest mean gap was reported in hospitals affiliated to Golestan University of Medical Sciences in northern Iran (23). Meta-analysis or merged estimate of mean effects in the study reviewed 18 articles (CI 95%: 0.72; 1.00) was obtained at 0.86 (Figure 5).

## Discussion

The results showed that according to opinions of all patients, there was a gap between the current status and the desirable status in all five dimensions. It means that patients' expectations were higher than their perceptions. In terms of the gap between the current status (patient perceptions) and the desirable status (patients' expectations), responsiveness was ranked first (1.04), followed by assurance (0.99), empathy (0.95), reliability (0.91), respectively and physical or tangible factors (0.86), respectively.

In this regard, many studies in other countries have also reported quality gap from the opinion of patients. Among these studies, we can refer to the studies conducted by Caha (8) in Turkey, Yesilada and Direktör (13) in Cyprus, Aldarmahi et al. (10) in Saudi Arabia, and Manulik et al. (12) in Poland the results of all of which were in line with those of our study. However, there are studies that suggest lack of quality gap and lack of match between patients' perceptions and expectations, including the studies conducted by Kalaja et al. (14) in Albania and Madan and Goel (15) in Indian hospitals where their results were inconsistent with those of our study. Also, results of our study concerning the five dimensions of quality and the analysis of the results of the reviewed studies in terms of patients' perceptions show that the largest gap belongs to the dimension of responsiveness. In the study conducted by Qolipour et al. (21) at Ahvaz Jundishapur University of Medical Sciences in southwestern Iran, in a study conducted by Razmjoee et al. in Shiraz University of Medical Sciences in Central Iran, in a study conducted by Haghshenas et al. (25) in Tehran University of Medical Sciences, in a study conducted by Tabibi et al. (35) in Tehran University of Medical Sciences in northern Iran, in a study conducted by Aghamolaei et al. at Hormozgan University of Medical Sciences in southern Iran (29), and in a study conducted by Aldarmahi in Saudi Arabia (10), the highest quality gap was reported in the responsiveness dimension which was consistent with the results of our study.

Also, in the studies conducted by Razlansari et al. in Kermanshah teaching hospitals in western Iran (30), AbolghasemGorji et al. in Imam Khomeini teaching hospital in Tehran University of Medical Sciences (31), Ameryoun et al. in selected hospitals in northern Iran (32), Mohammadi and Mohammadi at Zanjan Health Centers in central Iran (33), Jenaabadi et al. in Zahedan in eastern Iran (40), Havasbeigi et al. in public hospitals of Ilam and Kermanshah provinces in western Iran (37), and Nekoei-Moghadam and AmirEsmaeili in hospitals affiliated to Kerman University of Medical Sciences in central Iran (39), responsiveness dimension was ranked second. As patients in addition to physical diseases suffer mental and psychological disorders and anxiety due to physical diseases, they need more responsiveness from the clinical and administrative staff of the hospital so that their treatment course is completed and they achieve mental relaxation after the treatment. For example, the availability of the medical team is one of the dimensions of responsiveness. If those who are in charge of health care perform the treatment process appropriately, the patient will feel that the medical team is available and that responsiveness will be at the desirable level.

The medical education system of Iran has been integrated with the healthcare system in 1985 and now both are performed under the supervision of the Ministry of Health and Medical Education (with the aim of compensating for the severe shortage of health professionals, increasing the student acceptance capacity to compensate for the existing shortcomings and bilateral interaction of the medical education and health systems and ultimately the equitable access for all people to high quality healthcare services) (43). However, in almost all countries of the world, the medical education system and the healthcare provision system are working independently and under the supervision of two independent ministries. The results of our study showed that the quality of healthcare services provided in hospitals affiliated to Iran University of Medical Sciences was lower than the patients' expectations. Also, in a recent systematic review and meta-analysis study conducted to examine the quality of educational services provided by universities of medical sciences affiliated to the Ministry of Health of Iran, results showed that the quality of educational services was lower than the expectations of students studying at these universities (42).

## Limitations

Only systematic statistical analysis (meta-analysis) based on the SERVQUAL Evaluation Model was used in the present study to assess the quality of healthcare services provided in hospitals and health centers affiliated to medical science universities. Therefore, it is recommended to use other methods, especially objective and non-quantitative methods to obtain more accurate results.

## Conclusion

The results showed that patients' expectation level in university hospitals was higher than their perception and the highest gap belonged to the dimension of responsiveness. Responsiveness is related to areas such as providing appropriate and timely services, provider reliability, and good communication between staff or physicians and patients. The importance of these areas highlights the need for taking steps toward providing more appropriate and high-quality services. Services should be provided in such way that they reduce the gap between patients' perceptions and expectations and patients' perceptions of services should be enhanced. Further studies focusing on patient-centered solutions to improve the quality of services in the desired areas seem to be necessary. Therefore, it is recommended for “accreditation” and “clinical governance” units of the Ministry of Health and Medical Education of Iran to monitor periodically the quality of hospitals while focusing on patients' and students' satisfaction and pay attention to the dimensions that have the highest quality gap.

## Data Availability Statement

The original contributions presented in the study are included in the article/supplementary material, further inquiries can be directed to the corresponding author/s.

## Author Contributions

AG and AT designed research. AG conducted research, analyzed data, wrote manuscript, and he had primary responsibility for final content. All authors read and approved the final manuscript.

## Conflict of Interest

The authors declare that the research was conducted in the absence of any commercial or financial relationships that could be construed as a potential conflict of interest.

## Publisher's Note

All claims expressed in this article are solely those of the authors and do not necessarily represent those of their affiliated organizations, or those of the publisher, the editors and the reviewers. Any product that may be evaluated in this article, or claim that may be made by its manufacturer, is not guaranteed or endorsed by the publisher.
